# Hypersensitivity pneumonitis

**DOI:** 10.1186/1750-1172-1-25

**Published:** 2006-07-03

**Authors:** Yves Lacasse, Yvon Cormier

**Affiliations:** 1 Centre de Pneumologie, Université Laval, Hôpital Laval, 2725 Chemin Ste-Foy, Ste-Foy, Quebec, G1V 4G5, Canada

## Abstract

Hypersensitivity pneumonitis (HP) is a pulmonary disease with symptoms of dyspnea and cough resulting from the inhalation of an antigen to which the subject has been previously sensitized. The incidence of HP is unknown. A population-based study estimated the annual incidence of interstitial lung diseases as 30:100,000 and HP accounted for less than 2% of these cases. The diagnosis of HP can often be made or rejected with confidence, especially in areas of high or low prevalence respectively, using simple diagnostic criteria. Chest X-rays may be normal in active HP; High Resolution Computed Tomography is sensitive but not specific for the diagnosis of HP. The primary use of pulmonary function tests is to determine the physiologic abnormalities and the associated impairment. Despite the pitfalls of false positive and false negatives, antigen-specific IgG antibodies analysis can be useful as supportive evidence for HP. Bronchoalveolar lavage plays an important role in the investigation of patients suspected of having HP. A normal number of lymphocytes rules out all but residual disease. Surgical lung biopsy should be reserved for rare cases with puzzling clinical presentation or for verification the clinical diagnosis when the clinical course or response to therapy is unusual. Being an immune reaction in the lung, the most obvious treatment of HP is avoidance of contact with the offending antigen. Systemic corticosteroids represent the only reliable pharmacologic treatment of HP but do not alter the long-term outcome. The use of inhaled steroids is anecdotal. Treatment of chronic or residual disease is supportive.

## Disease name and synonyms

Hypersensitivity pneumonitis – Extrinsic allergic alveolitis

## Definition

Hypersensitivity pneumonitis (HP) is a pulmonary disease with symptoms of dyspnea and cough resulting from the inhalation of an antigen to which the subject has been previously sensitized. Acute and subacute HP represent the most active forms of the disease which may become chronic while remaining progressive. HP may also evolve to end-stage lung [1]. The diagnosis of HP has most often relied on an array of nonspecific clinical symptoms and signs developed in an appropriate setting [2], with the demonstration of interstitial markings on chest radiographs, serum antibodies against offending antigens, a lymphocytic alveolitis on bronchoalveolar lavage (BAL), and/or a granulomatous reaction on lung biopsies.

## Etiology

A wide spectrum of antigens may trigger the disease. These antigens have often led to a graphic and most descriptive nomenclature detailed in several case reports. A complete review of these antigens is beyond the scope of this article. The offending antigens can be classified in five broad categories represented by disease prototypes (Table 1).

**Table 1:** Prototypes of hypersensitivity pneumonitis according to major classes of antigens

**Class of antigens**	**Specific antigen**	**Disease**
Bacteria	*Saccharopolyspora rectivirgula*	Farmer's lung
Fungus	*Trichosporon cutaneum*	Summer-type HP
Mycobacteria	*Mycobacterium avium intracellulare*	Hot-tub lung
Proteins	Altered pigeon serum (probably IgA)	Pigeon breeder's disease
Chemical products	Diphenylmethane diisocyanate (MDI)	MDI HP

## Epidemiology

Like most interstitial lung diseases, HP is a rare disease. In a population-based study, the estimated annual incidence of interstitial lung disease was reported as 30 per 100,000 [3]. In that study, HP accounted for less than 2% of the incident cases. The study was conducted in New Mexico, a dry environment that is not propitious to the development of many forms of HP. In the HP Study [4], 30% of the 661 patients included in this prospective multi-center cohort had HP. This cohort study included consecutive adult patients presenting with a pulmonary syndrome for which active HP was considered in the differential diagnosis.

Over the last two or three decades, the difficulties in studying the epidemiology of HP have been illustrated by studies of the incidence or prevalence of farmer's lung. Definite conclusions have been elusive because of methodological issues including study design and the definition of farmer's lung [5-7]. Most studies used cross-sectional surveys in order to determine the prevalence of farmer's lung or that of associated conditions such as the presence of precipitating antibodies against offending antigens. Few, if any, real cohort studies have been published on the incidence of the disease [8-10]. An even more important factor has been the lack of a consistent definition of farmer's lung. Epidemiological reports based on cases admitted to a hospital where a definite diagnosis can be made using chest radiographs, computed tomography, BAL and/or lung biopsies are likely to identify the most severe cases only and thus underestimate the true prevalence of the disease. In addition, important differences have been observed in the classification of respiratory diseases among farmers by clinicians from different European countries [11]. In a survey of final diagnostic classifications on hospital discharge, 73% of cases of HP were erroneously classified [12]. Finally, fluctuations in the prevalence of farmer's lung have been related to a greater diagnostic suspicion attributable to ongoing epidemiological surveys [13]. Despite these methodological limitations, several studies gave consistent results allowing the prevalence of farmer's lung in exposed farmers to be estimated at between 0,5 and 3% [14-19].

The difficulties in establishing the incidence and prevalence of HP are further complicated by geographic variables, including climatic conditions and, in the case of farmer's lung, farming practices. Sex differences for both HP and seropositivity are likely to represent differences in exposure to offending antigens [20-22]. Genetic markers have generally failed to confirm hereditary risk factors for HP [23-33].

## Diagnostic criteria/Clinical presentation

A number of diagnostic criteria recommendations for HP have been published [34-37] (Table 2). The most widely used are those from Richerson and colleagues [35]. None of these sets of criteria have been validated. Their diagnostic accuracy is therefore unknown. They correspond in effect to definitions of the disease.

**Table 2:** Proposed diagnostic criteria for hypersensitivity pneumonitis for clinical purposes

**Author**	**Major criteria**	**Minor criteria**
Terho [10]	1. exposure to offending antigens (revealed by history aerobiological or microbiological investigations of the environment, or measurements of antigen-specific IgG antibodies) 2. symptoms compatible with HP present and appearing or worsening some hours after antigen exposure; 3. lung infiltrations compatible with HP visible on chest X-ray	1. basal crepitant rales 2. impairment of the diffusing capacity 3. oxygen tension (or saturation) of the arterial blood either decreased at rest, or normal at rest but decreased during exercise 4. restrictive ventilation defect in the spirometry 5. histological changes compatible with HP 6. positive provocation test whether by work exposure or by controlled inhalation challenge
Richerson *et al*. [35]	1. the history and physical findings and pulmonary function tests indicate an interstitial lung disease 2. the X-ray film is consistent 3. there is exposure to a recognized cause 4. there is antibody to that antigen	
Cormier *et al*. [36]	1. appropriate exposure 2. inspiratory crackles 3. lymphocytic alveolitis (if BAL is done) 4. dyspnea 5. infiltrates on chest radiographs or High Resolution Computed Tomography (HRCT)	1. recurrent febrile episodes 2. decreased Diffusing Capacity Test (DLCO) 3. precipitating antibodies to HP antigens 4. granulomas on lung biopsy (usually not required) 5. improvement with contact avoidance or appropriate treatment
Schuyler *et al*. [37]	1. symptoms compatible with HP 2. evidence of exposure to appropriate antigen by history or detection in serum and/or BAL fluid antibody 3. findings compatible with HP on chest radiograph or HRCT 4. BAL fluid lymphocytosis 5. pulmonary histologic changes compatible with HP 6. positive «natural challenge»	1. bibasilar rales 2. decreased DLCO 3. arterial hypoxemia, either at rest or during exercise

Others have developed prediction rules (*i.e*., clinical tools that quantify the contribution of various components of the history, physical examination and basic laboratory results to the diagnosis in an individual patient [38]) for periodic surveillance in high-risk workers or case finding in outbreaks of HP [39-41]. Although these rules are meant to be sensitive (*i.e*., able to detect most cases of work-related HP), it is likely that their specificity is limited in work environments with a high prevalence of other respiratory diseases. Little information is provided for their accuracy.

### The HP study

We recently addressed the issue of the clinical diagnostic criteria of HP in a prospective multi-centre cohort study [4]. Its objective was to develop a clinical prediction rule for the diagnosis of active HP. Such a rule aims at helping clinicians to arrive at a more accurate estimate of probability of HP and decide whether further investigation is needed to either rule in or rule out HP.

Consecutive adult patients presenting with a pulmonary syndrome for which active HP was considered in the differential diagnosis were included in this study. This cohort thus included a wide range of patients presenting for the investigation of a suspected interstitial lung disease, including patients with HP (the «cases») and patients without HP (the «controls»). Regression analyses identified six significant predictors of active HP (Table 3).

**Table 3:** Significant predictors of hypersensitivity pneumonitis*

**Variables**	**Odds ratio**	**Confidence interval (95%)**
Exposure to a known offending antigen	38.8	11.6 – 129.6
Positive precipitating antibodies	5.3	2.7 – 10.4
Recurrent episodes of symptoms	3.3	1.5 – 7.5
Inspiratory crackles	4.5	1.8 – 11.7
Symptoms 4–8 hours after exposure	7.2	1.8 – 28.6
Weight loss	2.0	1.0 – 3.9

* From Lacasse *et al*. [4], with permission.

The clinical prediction model produced an equation expressing the probability of HP as a function of the statistically significant variables. From this equation, we constructed a table of probability for combinations of predictors (Table 4). In clinical practice, the best diagnostic strategy will depend on the probability of HP determined from Table 4.

**Table 4:** Probability (%) of having hypersensitivity pneumonitis*

Exposure to a known offending antigen	Recurrent episodes of symptoms	Symptoms 4–8 hours after exposure	Weight loss	Crackles			
				
				+		-	
				
				Serum precipitins		Serum precipitins	
				
				+	-	+	-
+	+	+	+	98%	92%	93%	72%
+	+	+	-	97%	85%	87%	56%
+	+	-	+	90%	62%	66%	27%
+	+	-	-	81%	45%	49%	15%
+	-	+	+	95%	78%	81%	44%
+	-	+	-	90%	64%	68%	28%
+	-	-	+	73%	33%	37%	10%
+	-	-	-	57%	20%	22%	5%
-	+	+	+	62%	23%	26%	6%
-	+	+	-	45%	13%	15%	3%
-	+	-	+	18%	4%	5%	1%
-	+	-	-	10%	2%	2%	0%
-	-	+	+	33%	8%	10%	2%
-	-	+	-	20%	4%	5%	1%
-	-	-	+	6%	1%	1%	0%
-	-	-	-	3%	1%	1%	0%

* All the predictors are dichotomous variables; - indicates absent; +, present; from Lacasse *et al*. [4], with permission.

For instance, in a farmer presenting with recurrent episodes of respiratory symptoms, inspiratory crackles and testing positive for the corresponding precipitating antibodies, the probability of HP would be 81% (Table 4). Another patient presenting with progressive dyspnea and inspiratory crackles as the unique criteria of HP would have a probability of HP of less than 1%. Further investigation would be mandated only in the former. Typical findings of an alveolar lymphocytosis and/or bilateral ground-glass opacities on HRCT in the former patient would secure the diagnosis of HP, without resorting to surgical lung biopsy. HP would be confidently ruled out in the latter and the investigation oriented towards another diagnosis.

### Classification of HP

Much confusion still surrounds the classification of HP. Its clinical presentations have classically been defined as acute, subacute and chronic [35]. In the **acute form**, influenza-like symptoms often predominate, consisting of chills, fever, sweating, myalgias, lassitude, headache, and nausea that begin 2 to 9 hours after exposure, peak typically during 6 and 24 hours, and last from hours to days. Respiratory symptoms such as cough and dyspnea are common but not universal. The **subacute form **may appear gradually over several days to weeks, is marked by cough and dyspnea, and may progress to severe dyspnea and cyanosis, leading to urgent hospitalization. The **chronic form **has an insidious onset over a period of months, with increasing cough and exertional dyspnea. Fatigue and weight loss may be prominent symptoms.

The distinction between the stages of HP is often difficult as they likely represent different manifestations of a single disease that may be related more to the pattern of antigen exposure than to the offending antigen itself. This statement is supported by the finding of considerable overlap in the clinical manifestations of patients with farmer's lung (usually considered as the prototype of acute HP) and those with pigeon breeder's or bird fancier's diseases (the prototypes of subacute and chronic HP, respectively) [42]. Also, chronic HP may still be active and progressive. Others have suggested a classification that takes into account the progression of the disease (acute intermittent, acute progressive, chronic progressive, chronic nonprogressive) that can only be assessed retrospectively [1,5]. For practical purposes, we have already suggested to consider HP patients as having either active or residual disease, the latter representing late emphysematous or fibrotic sequelae of the disease in which the typical alveolar lymphocytosis of active HP has disappeared [4].

### Chest radiology

• Chest X-ray: Chest radiography is often the initial step in the investigation of a patient presenting with a pulmonary syndrome suggestive of HP. The first objective of chest X-rays is not to rule in HP but rather to rule out other diseases for the patient's illness. In acute HP, one expects to find groundglass infiltrates, nodular and/or striated patchy opacities [43,44]. The distribution of these infiltrates is usually diffuse but often sparing the bases in the subacute form [45]. A variety of different distributions have been described [46,47]. None of these findings are specific to HP. Up to 20% of individuals with acute HP have normal chest X-rays [48].

• CT scanning: Our ability to judge the usefulness of High Resolution Computed Tomography (HRCT) in HP is limited by the small number of cases studied. Table 5 summarizes selected reports of HRCT findings according to the phase of disease. The described patterns are not specific but suggest that HP may be considered in the differential diagnosis when present. For instance, groundglass opacities can be seen in a variety of other diseases including desquamative interstitial pneumonitis, *Pneumocystis carinii *pneumonia, bronchiolitis obliterans with organizing pneumonia, bronchoalveolar carcinoma, alveolar proteinosis, and alveolar hemorrhage. Conversely, when groundglass opacities are associated with poorly defined, centrilobular micronodules and mosaic attenuation or expiratory air trapping, the diagnosis of HP is further supported, but such an association is rare.

**Table 5:** High-resolution computed tomography findings in hypersensitivity pneumonitis

**Stage of disease**	**References**	**Sample size**	Findings *
*Acute*	Cormier *et al*. [49]	N = 20 (farmer's lung)	• ground-glass opacities • micronodules • mosaic perfusion • emphysema • honeycombing • mediastinal lymphadenopathis
*Subacute*	Hansell *et al*. [50]	N = 17 (including 9 with pigeon breeder's disease and 4 with farmer's lung)	• generalized increase in attenuation of the lung • nodular pattern • reticular pattern • patchy air space opacification
	Remy-Jardin *et al*. [51]	N = 21 (pigeon breeder's disease)	• micronodular pattern (< 5 mm in diameter) • ground-glass attenuation • emphysematous changes • honeycombing
*Chronic*	Adler *et al*. [52]	N = 16 (antigen = ?)	• fibrosis • ground-glass attenuation • nodules
	Remy-Jardin *et al*. [51]	N = 24 (pigeon breeder's disease)	• honeycombing • ground-glass attenuation • micronodules • emphysema

* The findings are ranked according to their decreasing order of prevalence in the study population.

### Pulmonary function tests

The primary use of pulmonary function tests is to determine the physiologic abnormalities and the associated impairment. The results of pulmonary function tests may also guide therapy by helping the clinician to select those for whom a treatment with corticosteroids may be justified. Pulmonary function tests have no discriminative properties in differentiating HP from other interstitial lung diseases [4].

The typical physiological profile of acute HP is a restrictive pattern with low DLCO [53]. In chronic disease, the pattern can be restrictive, but at least in farmer's lung, the most frequent profile is an obstructive defect resulting from emphysema [54]. The currently held belief is that a decreased DLCO is always present in HP [55]. Nevertheless, in the HP Study, 39 of the 177 patients (22%) in whom DLCO could be measured had normal results (defined as a DLCO 80% predicted) at the time of diagnosis [HP Study Group, unpublished data].

### Specific antibodies

The usefulness of most reports on the sensitivity and specificity of serum specific antibodies is limited by the inclusion of inappropriate controls, often healthy subjects. HP cannot be ruled in solely on the basis of positive antibodies or ruled out on the basis of negative antibodies. Many asymptomatic farmers (10%) and pigeon breeders (40%) have positive results [5,23,56] and many HP patients are negative for specific antibodies [57]. It is unclear if HP can occur in the absence of specific antibodies to the responsible allergen. False negatives could result from testing for inappropriate antigens.

Despite the pitfalls discussed above, specific antibodies analysis can be useful as supportive evidence. The results of the HP Study demonstrate that positive serum antibodies are a significant predictor of HP (odds ratio: 5.3; 95% CI: 2.7 – 10.4) [4]. Antigens available for testing in most centers included pigeon and parakeet sera, dove feather antigen, *Aspergillus sp*, *Penicillium*, *Saccharopolyspora rectivirgula*, and *Thermoactinomyces viridans*. These antigens cover most cases of HP including pigeon breeder's disease, bird fancier's lung, farmer's lung, and humidifier lung. The antigen *Trichosporon cutaneum *is also available in Japan for cases of summer-type HP [58]. The selection of antigens to be tested often needs to be determined locally according to the prevalent antigens [4,59]. In Eastern France, by using a panel of antigens really responsible for farmer's lung and not a classical standardized panel, serological tests showed a high rate of sensitivity and specificity [60].

Several methods for determination of precipitins or total IgG antibodies (immunodiffusion, immunoelectrophoresis, enzyme-linked immunosorbant assays [ELISA]) and different antigen preparations have been described [61,62]. ELISA is usually the preferred method. Unfortunately, even the ELISA technique lacks standardization [63].

### Inhalation challenge

Inhalation challenges to suspected environments, usually at the workplace, as well as specific provocation tests in controlled conditions have been described [64]. These tests lack standardization both in the inhalation protocols and in the criteria defining a positive response. Further studies are needed before recommending inhalation challenges in the diagnosis of HP.

### Bronchoalveolar lavage

BAL plays an important role in the investigation of patients suspected of having HP [65]. BAL can provide useful, supportive elements in the diagnosis of HP. A normal number of lymphocytes rules out all but residual disease [66]. However, the presence of an alveolar lymphocytosis does not, by any means, establish the diagnosis because asymptomatic, exposed individuals can also have increased numbers of lymphocytes in their BAL [67]. These individuals do not have subclinical HP, as confirmed by a 20-year follow-up [68]. Also many other diseases (including sarcoidosis, interstitial pneumonia associated with collagen vascular disease, silicosis, bronchiolitis obliterans with organizing pneumonia, HIV-associated pneumonitis and drug-induced pneumonitis) are characterized by an alveolar lymphocytosis [65]. Positive BAL findings (specially if the observed lymphocytosis is marked) [67,68] in a patient with interstitial lung disease of unknown origin should direct the clinician towards the possible diagnosis of HP [65].

As in the case of serum precipitins and inhalation challenge, the BAL technique lacks standardization. Lymphocyte subsets, especially the CD4/CD8 ratio and activation were previously thought to be helpful in differentiating HP from sarcoidosis. This is now challenged since the CD4/CD8 ratio in HP can be as high as that seen in sarcoidosis [71-73]. A low ratio would however support HP over sarcoidosis.

### Lung biopsy

The histopathology of HP has been well described [74-76]. In the acute stages, reports on open lung biopsies revealed features of interstitial lymphocytes infiltrates and fibrosis, edema, noncaseating granulomas, and bronchiolitis obliterans. Macrophages with foamy cytoplasm are also found within the alveolar space. In chronic stages, widespread fibrotic reaction is a prominent feature, often without predominant involvement of upper lobes with contraction. Even though emphysema was found at necropsy in chronic HP, it is only recently that emphysema has been recognized as a long-term complication of HP [54].

• Transbronchial biopsy: Hematoxylin-eosin-stained transbronchial biopsy is of limited usefulness for the diagnosis of farmer's lung [77].

• Surgical lung biopsy: The utility of surgical lung biopsy has most often been reported in terms of "diagnostic yield", *i.e*., the proportion of specific diagnoses obtained from the procedure. Whether the procedure alters the clinical management represents an important outcome. Several retrospective studies addressing these issues in series of patients with a variety of diffuse parenchymal diseases are available [78-89]. In selected reports, the results have been very heterogeneous: the diagnostic yield ranged from 34% to 100%; therapy was altered in 46% to 75% of the cases. This heterogeneity may stem from several factors, including the selection of candidates to open lung biopsy, the timing of the procedure along the course of the disease, as well as the expertise of the attending pathologist. The decision to submit a patient to open lung biopsy must be balanced against the associated morbidity. If HP is suspected, it has been our recommendation to reserve surgical lung biopsy for rare cases with puzzling clinical presentation or for verification the clinical diagnosis when the clinical course or response to therapy is unusual [36]. This recommendation is not based on evidence but emphasizes the limitations of surgical lung biopsy and the necessity of a thorough clinical investigation that comprises a high index of suspicion and a careful exposure history.

## Differential diagnosis

The differential diagnosis of HP is wide. The results of the HP Study illustrate this situation [4]. In this cohort study, consecutive adult patients presenting with a pulmonary syndrome for which active HP was considered in the differential diagnosis were included. The investigators had to classify each patient as HP or non-HP (*i.e*., control). The control group may be regarded as a set of lung diseases that must be distinguished from HP (Table 6).

**Table 6:** Distribution of diagnoses in the HP Study [4]

**Diagnosis**	**Number of patients**
**Hypersensitivity pneumonitis**	**199**
Pigeon breeder's/bird fancier's disease	132
Farmer's lung	38
Humidifier lung	3
Suberosis	2
Summer type HP	2
Various exposures to fungi	19
HP of unknown origin	3
**Controls**	**462**
Idiopathic interstitial pneumonia *	226
Sarcoidosis	52
Interstitial disease associated with collagen vascular disease	35
Drug induced pulmonary disease	26
Bronchiolitis obliterans (with our without organizing pneumonia)	25
Unspecified interstitial lung disease †	26
Infectious pneumonia	11
Histiocytosis X	10
Asthma	6
Silicosis	5
Eosinophilic pneumonia	5
Normal lung	4
Bronchoalveolar carcinoma/carcinomatous lymphangitis	4
Residual HP ‡	3
Residual HP ‡	3
Organic dust toxic syndrome	3
Lymphocytic interstitial pneumonia	2
Pulmonary edema (heart failure)	2
Radiation pneumonitis	2
Miscellaneous §	13
**TOTAL**	**661**

* includes patients with the clinical diagnosis of idiopathic pulmonary fibrosis, and those with the pathological diagnoses of usual, desquamative, respiratory bronchiolitis, acute and non-specific interstitial pneumonia;

† includes patients in whom no specific diagnosis could be reached but in whom HP was excluded on the basis of BAL;

‡ includes late emphysematous or fibrotic sequelae of HP in which the typical alveolar lymphocytosis of active HP has disappeared;

§includes single cases of alveolar hemorrhage, anthracosis, berylliosis, Churg-Strauss syndrome, diffuse panbronchiolitis, hepato-pulmonary syndrome, HIV-associated nonspecific interstitial pneumonia, necrotizing sarcoid granulomatosis, pulmonary amyloidosis, alveolar proteinosis, crack lung, Pneumocystis carinii pneumonia, and Wegener's granulomatosis.

## Management

### Treatment

As HP is a hyper immune reaction of the lung, the most obvious treatment is avoidance of contact with the antigen. Systemic corticosteroids represent the only recognised pharmacologic treatment for HP. The best available evidence comes from a unique randomized placebo-controlled trial [90]. In this trial, 36 patients with acute farmer's lung were randomized to receive either 40 mg of Prednisolone tapering over 8 weeks or placebo. All patients were instructed to avoid contact with the farm during the drug trial. After one month of treatment, there was no difference in FEV1, FVC and pO2 between the two groups. However, a small but significant difference in DLCO was observed. Corticosteroids had no beneficial effect on the long-term (5-year) prognosis however. The results of that trial confirmed other observations from controlled but non-randomized trials [91,92] and case series: corticosteroids hasten the recovery from the acute stage of HP, but have no beneficial effect on long-term prognosis. The decision to treat with corticosteroids may be guided by the severity of symptoms and physiologic abnormalities [93]. The use of inhaled steroids is anecdotal [94]. The treatment of chronic or residual disease is supportive.

### Prevention

In high-risk environments (such as farming activities), education may prevent respiratory problems [95]. Ideally, all farmers should be informed of the hazards of exposure to barn dust and encouraged to use adequate preventive measures. For practical purposes, however, major preventive measures (such as mask wearing, increasing barn ventilation, avoiding the barn when the animals are feeding) cannot be recommended for primary prevention and are usually reserved for individuals with past history of HP [96].

## Unresolved questions

A recent workshop of the National Heart, Lung, and Blood Institute and the Office of Rare Diseases identified several areas for future clinical research in HP [97]. These include, among others, (1) the need for a better documentation of its incidence and prevalence; (2) the identification of genetic and environmental risk factors that affect its occurrence and natural history; (3) the validation of biomarkers of both exposure and disease; (4) the definition of its natural history; (5) the development of a battery of standardized antigens known to cause HP that should be available to clinicians and researchers for use in both the diagnosis and investigations of pathogenesis.
